# MicroRNA-300 Inhibits the Proliferation and Metastasis of Cervical Cancer Cells via Posttranscriptional Suppression of G Protein-Coupled Receptor 34 (GPR34)

**DOI:** 10.1155/2021/2669822

**Published:** 2021-12-14

**Authors:** Mei Wang, Ying Tian, Lin Miao, Wenxia Zhao

**Affiliations:** ^1^Department of Obstetrics and Gynecology, Xinhua Hospital Chongming Branch, School of Medicine, Shanghai Jiao Tong University, Shanghai 202150, China; ^2^Department of Gynecology, First Hospital Affiliated to Harbin Institute of Technology, Harbin, Heilongjiang 150010, China; ^3^Department of Obstetrics and Gynecology, Fourth People's Hospital, Shanghai 200080, China

## Abstract

Cervical cancer is one of the dominant gynecological disorders which has poor prognosis and often diagnosed at advanced stages where it becomes nearly impossible to effectively manage this disorder. MicroRNA-300 (miR-300) has dual role in human tumorogenesis. However, characterization of its regulatory action has not been made in cervical cancer. The molecular role of miR-300 in cervical cancer was thus explored in the present study with prime focus on elucidating its mechanism of action. The results showed significant (*P* < 0.05) downregulation of miR-300 in cervical cancer. Overexpression of miR-300 in cervical cancer cells inhibited their proliferation in vitro by inducing apoptosis. Cervical cancer cells overexpressing miR-300 also showed decreased rates of migration and invasion. G protein-coupled receptor 34 (GPR34) was found to be the functional regulatory target of miR-300 in cervical cancer. GPR34 was found to be significantly (*P* < 0.05) overexpressed in cervical cancer tissues and cell lines. Silencing of GPR34 inhibited the growth of the cervical cancer cells. However, overexpression of GPR34 could prevent the tumor-suppressive effects of miR-300 on cervical cancer cells. Collectively, the results of the current study are indicative of the tumor-suppressive regulatory role of miR-300 in cervical cancer and suggestive of the potential therapeutic value of miR-300/GPR34 molecular axis.

## 1. Introduction

Cervical cancer is one of the prevalent gynecological malignancies affecting about half a million women and causing 2,50,000 deaths annually across the globe [1]. The genetic and epigenetic aberrations together with HPV infection are some of the commonly reported risk factors of cervical cancer [2]. Studies have suggested that during recent few years, the incidence of cervical cancer has worriedly increased in younger females [3]. Moreover, cervical cancer is associated with poor prognosis despite recent improvements in the diagnostic measures and screening strategies [4]. Higher proportion of cervical cancer patients showing distant metastasis together with the chances of posttherapeutic recurrence marks the crucial hindrances in effective management of this lethal disease [5, 6]. Only about 15% of the cervical cancer patients survive over a period of five years at advanced stages [7]. Therefore, it is necessary to formulate effective anticancer measures against cervical cancer for improved therapeutic results in future.

Micro-RNAs (miRs) are referred to as a heterogenous class of noncoding RNAs ranging in size from 20 to 25 nucleotides which recently have become the focus of research for their involvement in key cellular processes such as growth, development, and differentiation [8, 9]. miRs repress the expression of eukaryotic genes at the posttranscriptional level by binding to specific DNA sites in 3′UTR of their target genes [10]. According to an estimate, expression of more than 30% protein-coding genes is affected by miRs in humans [11]. Several types of human disorders including cancer are reportedly associated with increased or decreased expression levels of specific miRs [12]. Oncogenic miRs are upregulated in cancer, while those which act as tumor-suppressors are significantly downregulated [13]. MicroRNA-300 (miR-300) usually acts as tumor suppressor in number of human cancers such as lung and hepatocellular cancers [14, 15]. Nevertheless, miR-300 has been shown to have an oncogenic role in gastric cancer [16]. In human epithelial cancer, miR-300 has been shown to target Twist gene to suppress the epithelial to mesenchymal transition [17]. Similarly, miR-300 has been reported to inhibit the growth of the laryngeal squamous cell carcinoma [18, 19]. The reduced expression of miR-300 has also been reported to be a predictor of poor prognosis in laryngeal squamous cell carcinoma patients [20, 21]. However, the regulatory function of miR-300 has not yet been studied in cervical cancer. Against this backdrop, the present study was designed to decipher the role of miR-300 in cervical cancer via modulation of G protein-coupled receptor 34 (GPR34) expression.

## 2. Materials and Methods

### 2.1. Human Tissues and Cell Lines

A total of 30 cervical cancer and adjacent normal tissues were collected after obtaining written consents from the cervical cancer patients who went through surgery at Fourth People's Hospital, Shanghai, China. Tissue collection was made prior to application of radio/chemotherapies. The tissues collected were pathologically investigated to validate their clinical status by three independent pathologists. The human tissues were frozen in liquid nitrogen, and their long-term storage was performed at −80^o^C. The institutional ethics committee provided the approval for conducting the study under approval number PHS/62/2019.

A panel of human cervical cancer cell lines (CaSki, HeLa, HT-3, and ME-180) together with the normal cervical epithelial cells (HcerEpic) was acquired from The Cell Bank of Type Culture Collection of Chinese Academy of Sciences (Shanghai, China). The cell lines were grown in Eagle's minimum essential medium (ATCC, Manassas, USA), supplemented with 10% concentration of fetal bovine serum (FBS; Gibco) and 1% penicillin/streptomycin (Invitrogen, Carlsbad, CA, USA). A humidified incubator was used for maintenance of cell lines at 37 °C with 5% CO_2_.

miR-300 mimics (5′-UAUACAAGGGCAGACUCUCUCU-3′), miR-NC (5′-UUCUCCGAACGUGUCACGUTT-3′), si-GPR34 (5′-CAGTTTGGATCGCTATATA-3′), si-NC (′5-TTCTCCGAACGTGTCACGT-3′), pcDNA3.1 plasmid, and pcDNA-GPR34 were purchased from Guangzhou RiboBio (Beijing, China). These constructs were transfected into CaSki and HeLa cancer cells using Lipofectamine 3000 (Invitrogen) according to the manufacturer's instructions. The cells were harvested after 48 h of transfection at 80% confluence.

### 2.2. Gene Expression Study

Total RNA was extracted from cell lines and tissues with the help of TRIzol (Thermo Fisher Scientific, Waltham, MA, USA). The RNA was reverse transcribed into complementary DNA (cDNA) with the help of the GoScript™ Reverse Transcription System (Promega, Madison, WI, USA). With cDNA as template, qRT-PCR was performed using SYBR Green PCR Master Mix (Thermo Fisher Scientific, Waltham, MA, USA). The thermal cycling parameters were as follows: 95°C for 5 min, 40 cycles of 95°C for 30 s, 60°C. GADPH and U6 snRNA served as an endogenous control for GPR34 and miR-300, respectively. The references genes were selected after confirmation of their stable expression across all samples. The relative expression levels of miR-300 and GPR34 were quantified using the 2^−ΔΔCt^ method.

### 2.3. MTT and Colony Formation Assays

The proliferation of transfected CaSki and HeLa cancer cells was determined by MTT assay. Briefly, the cells were placed in a 96-well plate with 2 × 10^4^ cells per well and then incubated for 12, 24, 48, or 96 h at 37°C. Next, 20 *μ*L MTT solution (5 mg/mL; Sigma-Aldrich; Merck KGaA) was added to each well, and incubation was prolonged for 2 h. The wells were subsequently given 150 *μ*L DMSO, and finally, the absorbance was determined using spectrophotometer at 570 nm wavelength.

For clonogenic assay, 5000 transfected CaSki and HeLa cancer cells were plated into 6-well plates and cultured for two weeks at 37^o^C. The culture medium was changed every 3 days. Culturing was performed till the clones were visible in the wells. At this, the colonies were subjected to phosphate buffered saline (PBS) wash, fixed with 70% ethanol for 20 minutes, and subsequently stained using 0.05% crystal violet (Sigma-Aldrich). The colonies were then photographed, and relative colony formation was analyzed.

### 2.4. Annexin V-FITC/PI Apoptosis Assay

To study their apoptosis, the CaSki and HeLa cancer cells transfected with miR-300 mimics or miR-NC were processed for the Annexin V-FITC/PI staining method. In brief, the cancer cells (105 per well) were cultured in 24-well plates for 24 h at 37°C. This was followed by harvesting of cancer cells through centrifugation. The cells collected were then suspended in 250 *μ*L binding buffer and inoculated with 7.5 *μ*L each of Annexin V-fluorescein isothiocyanate (FITC) and propidium iodide (PI), serially. Afterwards, the cells were given multiple PBS washes, and their apoptosis was analyzed with the help of a flow cytometer (BD Biosciences, NJ, USA).

### 2.5. Western Blotting

Total proteins were isolated from tissues and cell lines by lysing them with ice‐cold RIPA lysis buffer. 45 micrograms of total proteins were subjected to electrophoresis on 10–12% SDS‐PAGE gels which were blotted onto the nitrocellulose membranes. The membranes were probed with specific primary antibodies at 4°C overnight. After being washed with PBS-T, the membranes were incubated with horseradish peroxidase-conjugated secondary antibodies at room temperature for 2 h. Membranes were again washed with TBST and finally scanned with Odyssey (Tanon, Shanghai, China). *β*-Actin was used as an internal expression control.

### 2.6. Migration and Invasion Assays

The transfected CaSki and HeLa cancer cells were seeded into a 6-well plate with an initial density of 2.5 × 105 cells/well. The cell surface was then wounded perpendicularly in a straight line using a sterile micropipette tip. The cellular debris was removed using PBS wash, and carved wound was photographed. The plate was subsequently incubated for 24 h at 37°C, and linear wound was again photographed with the help of a light microscope (Olympus Corporation, Tokyo, Japan) using 100X magnification.

After 48 hours of transfection, 2 × 10^4^ CaSki and HeLa cancer cells were placed into the upper chamber (8.0 *μ*m; Costar) separated from the underlying chamber with a porous polycarbonate membrane precoated with Matrigel solution. The lower chamber was given only the culture medium containing 10% FBS. This was followed by an incubation of 37°C for 24 h. The cells that had invaded into the lower chamber were fixed using 4% formaldehyde, and then, 0.1% crystal violet was used for their staining. The stained cells were observed under an inverted light microscope. Five microscopic fields were chosen at random and used for determining the relative percentage of cell invasion.

### 2.7. Bioinformatics Analysis and Luciferase Reporter Assay

The miR-300 was subjected to online bioinformatics through TargetScan Human 7.0 (https://www.targetscan.org/) and miRanda (https://www.microrna.org/microrna/) to predict its potential molecular targets. To authenticate the target prediction, the dual-luciferase reporter assay was performed. Briefly, 3′UTR of GPR34 with wild type (WT) or mutant (MUT) miR-300 binding site was cloned into the psiCHECK2 vector (Promega, Madison, WI, USA) downstream of the Renilla luciferase gene. The luciferase reporter constructs of GPR34 UTR (WT or MUT) were cotransfected with miR-300 mimics or miR-NC into CaSki cancer cells. The luciferase activity of transfected cells was then measured after 48 h of transfection with the help of the dual-luciferase reporter assay system (Promega) following the manufacturer's guidelines.

### 2.8. Immunohistochemical Staining

The immunohistochemical staining of GPR34 from human tissues was performed according to Im K et al., 2019 [17]. The analysis of the histochemical staining was performed by two independent pathologists blind to the study. The expression of GPR34 was considered be negative for no staining, positive but weak for light brown staining analysis, while it was described to be strong for dark brown staining.

### 2.9. Statistical Analysis

All experiments were performed using thrice in triplicate, and results were representative of mean ± SD. The GraphPad prism 7.0 software was used to perform the analysis of statistical data. The statistical differences were analyzed with Student's *t*-test and one-way ANNOVA. *P* < 0.05 was taken as the measure of a statistically significant difference.

## 3. Results

### 3.1. miR-300 Is Downregulated in Cervical Cancer

**Figure 1:**
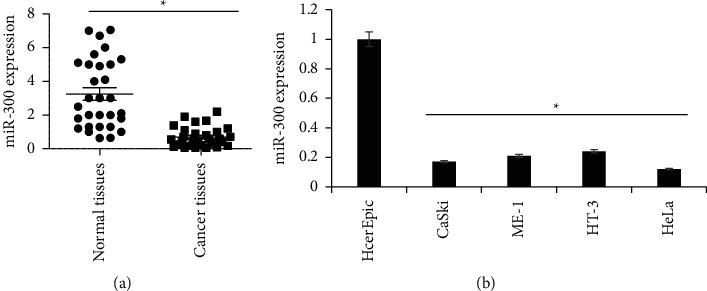
Cervical cancer exhibiting significant downregulation of miR-300. Relative expression analysis of miR-300 from (a) cervical cancer and normal matched tissues. (b) Cervical cancer cell lines (CaSki, HeLa, HT-3, and ME-180) and HcerEpic, normal cervical epithelial cells. The qRT-PCR study was performed using three replicates to analyze the miR-300 transcript levels. The experiments were performed thrice in triplicate and expressed as mean ± SD (^*∗*^*P* < 0.05).

RNA was isolated from human tissues (cancer and matched normal) and cell lines to analyze the expression of miR-300 using qRT-PCR. The results showed that cervical cancer tissues on average possessed significantly (*P* < 0.05) lower transcript abundance of miR-300 than the normal matched tissues (Figure 1(a)). In coherence with this, the cervical cancer cell lines (CaSki, HeLa, HT-3, and ME-180) were also shown to exhibit significantly (*P* < 0.05) lower miR-300 expression than HcerEpic, normal cervical epithelial cells (Figure 1(b)). CaSki and HeLa cell lines were used for molecular characterization of miR-300 role in cervical cancer owing to least miR-300 expression in them.

### 3.2. miR-300 Inhibits In Vitro Growth of Cervical Cancer Cells

**Figure 2:**
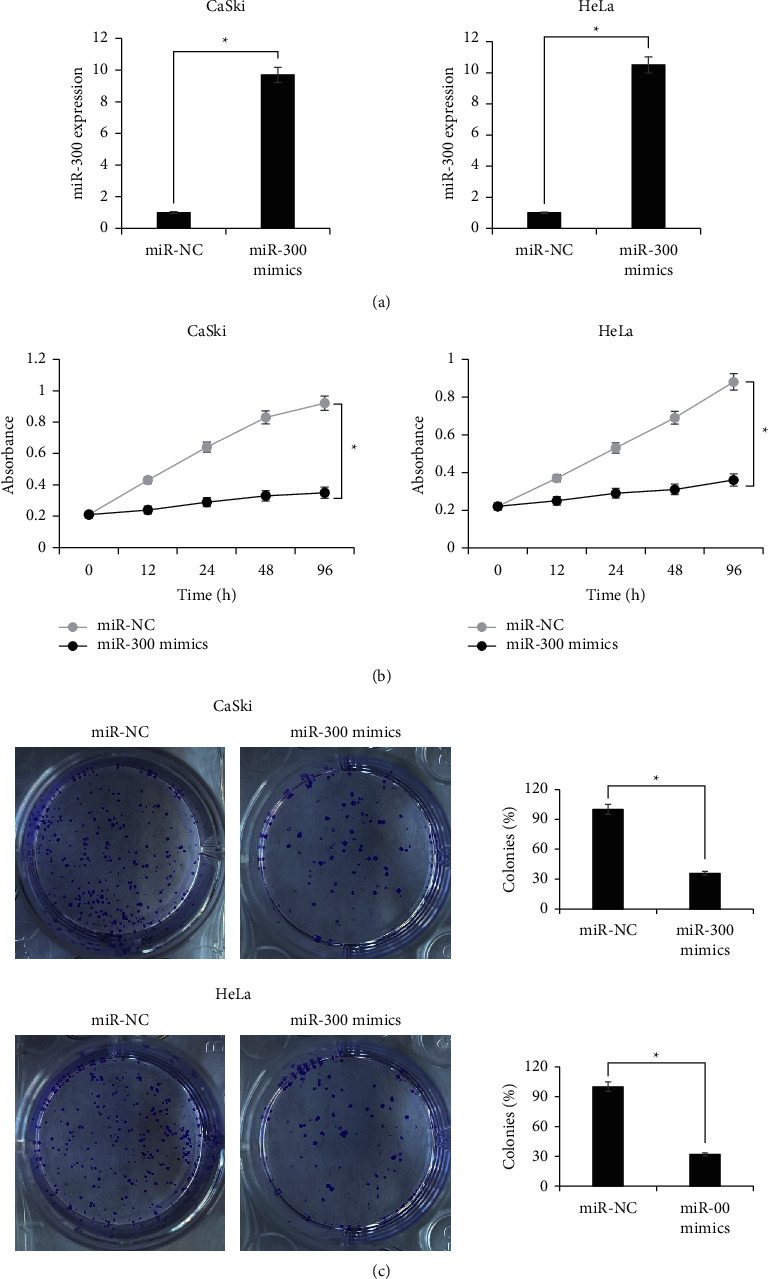
Cervical cancer cells overexpressing miR-300 exhibit loss of proliferation. (a) Transcript level analysis of miR-300 from CaSki and HeLa cancer cells transfected with miR-300 mimics or miR-NC. (b) MTT assay of CaSki and HeLa cancer cells transfected with miR-300 mimics or miR-NC. (c) Clonogenic assay of CaSki and HeLa cancer cells transfected with miR-300 mimics or miR-NC. The experiments were performed thrice in triplicate and values are presented as mean ± SD (^*∗*^*P* < 0.05).

To analyze the growth regulatory potential of miR-300 in cervical cancer, miR-300 was overexpressed in CaSki and HeLa cancer cells by transfecting them with miR-300 mimics. miR-NC transfected cancer cells served as the corresponding negative controls. miR-300 overexpression was confirmed by qRT-PCR. The CaSki and HeLa cells showed 7-fold upregulation of miR-300 with reference to the respective negative control cells (Figure 2(a)). MTT assay was then performed to analyze the cell proliferation at different time intervals. The proliferation of both CaSki and HeLa cells was found to be significantly (*P* < 0.05) inhibited by miR-300 overexpression (Figure 2(b)). Additionally, the colony formation from both CaSki and HeLa cancer cells was significantly (*P* < 0.05) decreased by miR-300 overexpression (Figure 2(c)). The relative colony formation was reduced by about 70% from CaSki and HeLa cancer cells overexpressing miR-300 when compared with that from the respective negative control cells. The results therefore are indicative of the negative growth regulatory role of miR-300 in cervical cancer.

### 3.3. Apoptosis Is Induced in Cervical Cancer Cells by miR-300

**Figure 3:**
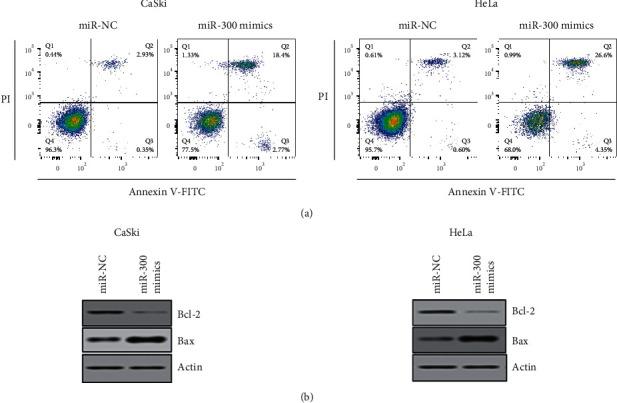
miR-300 overexpression inducing apoptosis in cervical cancer cells. (a) Annexin V-FITC/PI apoptosis assay of CaSki and HeLa cancer cells transfected with miR-300 mimics or miR-NC. (b) Western blotting of Bax and Bcl-2 proteins from CaSki and HeLa cancer cells transfected with miR-300 mimics or miR-NC. The experiments were performed twice in triplicate.

To find out the underlying mechanism responsible for the tumor-suppressive effect of miR-300 overexpression against the cervical cancer cells, the Annexin V-FITC/PI staining method combined with flow cytometry was used to study the apoptosis of CaSki and HeLa cancer cells overexpressing miR-300 using the respective miR-NC transfected cancer cells as corresponding negative controls. The flow-cytometric analysis showed that relative percentage of both early and late apoptosis increased considerably for both CaSki and HeLa cancer cells by miR-300 overexpression (Figure 3(a)). Collectively, the relative percentage of apoptosis increased from 3% to 21% for CaSki cells and 4% to 31% for HeLa cancer cells by miR-300 overexpression. Moreover, the intracellular levels of Bax protein were considerably upregulated, while that of Bcl-2 protein was downregulated by miR-300 overexpression in both CaSki and HeLa cancer cell lines (Figure 3(b)). The results thus suggest that miR-300 induces apoptosis in cervical cancer cells by modulating the intracellular levels of caspases and enhancing the Bax/Bcl-2 protein ratio.

### 3.4. miR-300 Decreases Cervical Cancer Cell Migration and Invasion and Inhibits Their Epithelial-Mesenchymal Transition

**Figure 4:**
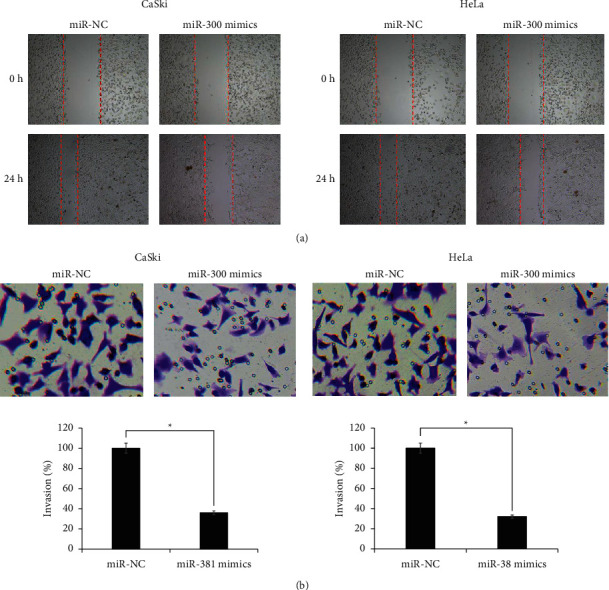
Overexpression of miR-300 inhibiting migration and invasion of cervical cancer cells. (a) Wound-healing assay of CaSki and HeLa cancer cells transfected with miR-300 mimics or miR-NC. (b) Transwell chamber assay of CaSki and HeLa cancer cells transfected with miR-300 mimics or miR-NC. The experiments were performed in thrice in triplicate and values are presented as mean ± SD (^*∗*^*P* < 0.05).

Whether miR-300 regulates the metastatic parameters of cervical cancer cells, the migration, and invasion of miR-300 overexpressing cervical cancer cells in relation to the respective negative control cells were studied through wound-healing and transwell chamber assays, respectively. Cervical cancer cell lines, CaSki and HeLa overexpressing miR-300, exhibited significantly (*P* < 0.05) lower migratory potential when compared with the respective negative control cells (Figure 4(a)). The migration of both cancer cell lines showed more than 40% decline once transfected with miR-300 mimics. In coherent with this, the invasion of CaSki and HeLa cancer cells also showed marked decline under miR-300 overexpression (Figure 4(b)). Both the cell lines overexpressing miR-300 showed 3-fold decline in their invasion rate in vitro with reference to the corresponding negative control cervical cancer cells. The results are suggestive of antimetastatic regulatory role of miR-300 in cervical cancer.

### 3.5. GPR34 Acts as the Posttranscriptional Target of miR-300 in Cervical Cancer

**Figure 5:**
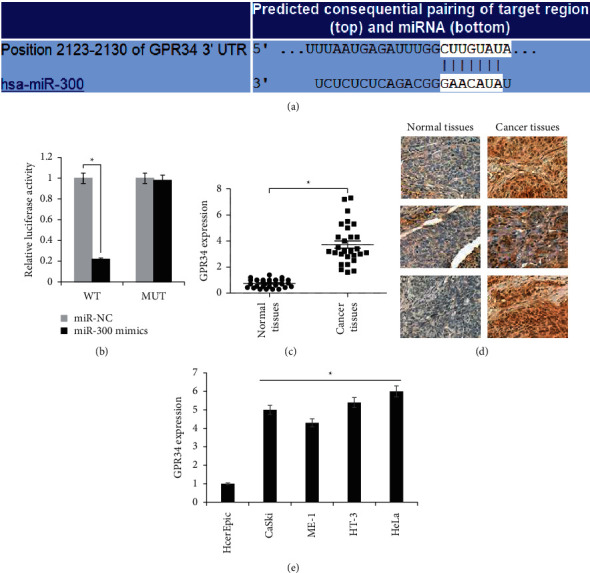
miR-300 targets GPR34 at the posttranscriptional level in cervical cancer. (a) In silico analysis for miR-300 target prediction. (b) miR-300 binding site identified in 3′UTR of GPR34. (c) Dual-luciferase assay of miR-300 with 3′UTR (WT or MUT) of GPR34 in CaSki cancer cells. (d) Relative expression analysis of miR-300 from cervical cancer and normal matched tissues. (e) Immunohistochemical staining analysis of GPR34 from four pairs of cervical cancer and normal matched tissues. (f) Western blotting of GPR34 from cervical cancer cell lines (CaSki, HeLa, HT-3, and ME-180) and HcerEpic, normal cervical epithelial cells. The experiments were performed in thrice triplicate and values are presented as mean ± SD (^*∗*^*P* < 0.05).

The online in silico analysis was used to analyze the regulatory targets of miR-300 (Figure 5(a)). GPR34 was indicated as the specific molecular target of miR-300, and the latter was predicted to bind the specific binding sequence in the 3′UTR of GPR34 (Figure 5(a)). The 3′UTR stretch of GPR34 was thus cloned with wild type (WT) or mutant (MUT) miR-300 binding site to generate the respective luciferase reporter construct which was then cotransfected with miR-300 mimics or miR-NC into CaSki cancer cells. Dual-luciferase assay was performed to assess the interaction of miR-300 with its predicted binding site in GPR34 UTR. The significant (*P* < 0.05) decline in the luciferase activity only with WT GPR34 3′UTR and miR-300 mimics cotransfection proved specific interaction of miR-300 with GPR34 UTR (Figure 5(b)). More support was gained from the expression analysis of GPR34 from cervical cancer and normal matched tissues. The qRT-PCR showed that cervical cancer tissues possess significantly (*P* < 0.05) higher transcript levels of GPR34 than the normal matched cervical tissues (Figure 5(c)). The immunohistochemical staining analysis of GPR34 from paired cancer and normal tissues from four cervical cancer patients also confirmed the upregulation of GPR34 in cervical cancer tissues (Figure 5(d)). As expected, the transcript levels of GPR34 were significantly (*P* < 0.05) higher in all the cervical cancer cell lines (CaSki, HeLa, HT-3, and ME-180) in comparison to the HcerEpic cervical epithelial cells (Figure 5(e)). The results of miR-300 interaction and expression analysis of GPR34 are indicative of posttranscriptional targeting of miR-300 in cervical cancer.

### 3.6. GPR34 Acts the Modulator of miR-300 Action in Cervical Cancer

**Figure 6:**
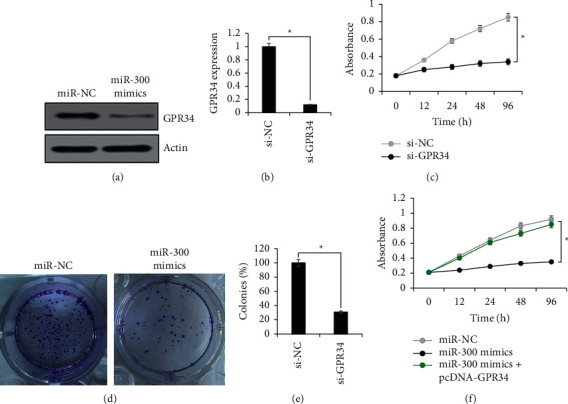
GPR34 modulating miR-34 molecular role in cervical cancer. (a) Western blotting of GPR34 from CaSki cancer cells transfected with miR-300 mimics or miR-NC. (b) Relative expression analysis of GPR34 from CaSki cancer cells transfected with si-GPR34 or si-NC. (c) MTT proliferation assay of CaSki cancer cells transfected with si-GPR34 or si-NC. (d) Clonogenic assay of CaSki cancer cells transfected with si-GPR34 or si-NC. (e) MTT proliferation assay of CaSki cancer cells transfected with miR-300 mimics, miR-NC, or miR-300 mimics plus pcDNA-GPR34. The experiments were performed in thrice in triplicate and values are presented as mean ± SD (^*∗*^*P* < 0.05).

The protein expression of GPR34 was shown to be significantly (*P* < 0.05) repressed by miR-300 overexpression (Figure 6(a)). In order to appraise the functional aspect of this posttranscriptional repression of GPR34 by miR-300, GPR34 was transiently downregulated in CaSki cancer cells, and GPR34 silencing was confirmed by qRT-PCR (Figure 6(b)). MTT assay showed that GPR34 downregulation in cervical cancer cells led to significant (*P* < 0.05) decrease in their in vitro proliferation rate at indicated culture intervals (Figure 6(c)). Similarly, the CaSki cancer cells downregulating GPR34 showed remarkably lower colony formation as that of the negative control cells (Figure 6(d)). Besides, the upregulation of GPR34 in CaSki cells overexpressing miR-300 attenuated the antiproliferative effects of miR-300 overexpression (Figure 6(e)). The results are thus conclusive that miR-300 functionally targets GPR34 in cervical cancer to exert its tumor-suppressive role.

## 4. Discussion

The process of tumorogenesis is very intricate and driven through genetic/epigenetic molecular irregularities which induce the cellular reprogramming to mark the onset of cancer hallmarks reflecting the development and subsequent propagation of malignant disorder [22]. Although, the protein-coding genes have attained comparatively much more research focus for their specific role in human cancers, but due to the possession of around 97% nonprotein-coding DNA sequences by human genome, the researchers have now shifted their focus towards this DNA dark matter for its possible involvement in regulating the carcinogenesis. Of the nonprotein-coding transcripts, the micro-RNAs (miRs) are being extensively studied for their cancer-specific role which not only has led to significant increase in our basic understanding of human cancer but has also enlightened the possibility of utilizing miRs as vital prognostic and therapeutic agents against this deadly disorder [23]. miRs have been shown to play oncogenic or tumor-suppressive role in human cancers to regulate cancer growth, propagation, and dissemination [24]. Several miRs like microRNA-300 (miR-300) reportedly exhibit dual regulatory role in cancer growth and progression [14–16]. However, miR-300 has not yet been evaluated for its specific molecular function in one of the dominant gynecological malignancies, the human cervical cancer.

The results of present study showed that miR-300 exhibits significant downregulation in cervical cancer tissues and cell lines which coincides with its expression in other human cancers such as glioma and bladder cancer [25, 26]. The inhibition of cervical cancer cell proliferation and clonogenic potential by miR-300 overexpression was suggestive of tumor-suppressive function of miR-300 in cervical cancer. Previously, proapoptotic molecular potential of miR-300 was shown to mediate the growth inhibitory action of miR-300 against pancreatic cancer cells [27]. The results of the present study showed that cervical cancer cells were inducted for apoptotic cell death by miR-300 via the increase in proapoptotic caspase proteins together with elevation of Bax/Bcl-2 protein ratio [28, 29]. Liang D et al. have recently reported that miR-300 inhibits the metastasis of osteosarcoma cells by targeting PTTG1 [30]. Furthermore, miR-300 has been shown to inhibit the epithelial-mesenchymal transition of pancreatic and oral squamous cancer cells [27, 31]. The inhibition of migration and invasion of cervical cancer cells by miR-300 supports the similar antimetastatic action of miR-300 against the cervical cancer. At the molecular level, miR-300 was shown to target GPR34 at the posttranscriptional level to exert its tumor-suppressive effects on cervical cancer cells. GPR34 belongs to G protein-coupled receptor family whose oncogenic behavior has been established by number of research studies [30, 31]. As evident from the expressional correlation between miR-300 and GPR34 in cervical cancer, downregulation of miR-300 might be one of the molecular factors responsible for GPR34 upregulation in this lethal malignancy whose oncogenic behavior could possibly be one of the key drivers of cervical cancer growth and progression. This proves the therapeutic usefulness of miR-300/GPR34 axis.

## 5. Conclusion

Collectively, the results of the current study are indicative of transcriptional repression of miR-300 in cervical cancer. Tumor-suppressive action of miR-300 was reflected by the fact that its overexpression not only inhibited apoptosis-driven cancer cell growth but also restricted the cancer cell migration and invasion. The tumor-suppressive role of miR-300 was confirmed to be mediated through GPR34 at the molecular level. The findings of the present are suggestive of the therapeutic potential of miR-300/GPR34 axis in the management of cervical cancer.

## Data Availability

The data used to support the findings of this study are available from the corresponding author upon request.
